# Gene Expression and Thiopurine Metabolite Profiling in Inflammatory Bowel Disease – Novel Clues to Drug Targets and Disease Mechanisms?

**DOI:** 10.1371/journal.pone.0056989

**Published:** 2013-02-21

**Authors:** Sofie Haglund, Sven Almer, Curt Peterson, Jan Söderman

**Affiliations:** 1 Division of Medical Diagnostics, Laboratory Medicine, Ryhov County Hospital, Jönköping, Sweden; 2 Division of Gastroenterology and Hepatology, Department of Clinical and Experimental Medicine, Faculty of Health Sciences, Linköping University, Linköping, Sweden; 3 Department of Gastroenterology, County Council of Östergötland/UHL, Linköping, Sweden; 4 Division of Drug Research, Department of Medicine and Health, Faculty of Health Sciences, Linköping University, Linköping, Sweden; University of Chicago, United States of America

## Abstract

**Background and Aims:**

Thiopurines are effective to induce and maintain remission in inflammatory bowel disease (IBD). The methyl thioinosine monophosphate (meTIMP)/6-thioguanine nucleotide (6-TGN) concentration ratio has been associated with drug efficacy. Here we explored the molecular basis of differences in metabolite profiles and in relation to disease activity.

**Methods:**

Transcriptional profiles in blood samples from an exploratory IBD-patient cohort (n = 21) with a normal thiopurine S-methyltransferase phenotype and meTIMP/6-TGN ratios >20, 10.0–14.0 and ≤4, respectively, were assessed by hybridization to microarrays. Results were further evaluated with RT qPCR in an expanded patient cohort (n = 54). Additionally, 30 purine/thiopurine related genes were analysed separately.

**Results:**

Among 17 genes identified by microarray-screening, there were none with a known relationship to pathways of purines/thiopurines. For nine of them a correlation between expression level and the concentration of meTIMP, 6-TGN and/or the meTIMP/6-TGN ratio was confirmed in the expanded cohort. Nine of the purine/thiopurine related genes were identified in the expanded cohort to correlate with meTIMP, 6-TGN and/or the meTIMP/6-TGN ratio. However, only small differences in gene expression levels were noticed over the three different metabolite profiles. The expression levels of four genes identified by microarray screening (*PLCB2*, *HVCN1*, *CTSS*, and *DEF8*) and one purine/thiopurine related gene (*NME6*) correlated significantly with the clinical activity of Crohn’s disease. Additionally, 16 of the genes from the expanded patient cohort interacted in networks with candidate IBD susceptibility genes.

**Conclusions:**

Seventeen of the 18 genes which correlated with thiopurine metabolite levels also correlated with disease activity or participated in networks with candidate IBD susceptibility genes involved in processes such as purine metabolism, cytokine signaling, and functioning of invariant natural killer T cells, T cells and B cells. Therefore, we conclude that the identified genes to a large extent are related to drug targets and disease mechanisms of IBD.

## Introduction

Ulcerative colitis (UC) and Crohn’s disease (CD) are chronic remitting and progressive inflammatory bowel diseases (IBD). Primarily, 5-aminosalicylic acid (5-ASA) and glucocorticosteroids are used in the treatment. Glucocorticosteroid dependent or refractory patients are eligible for immunomodulatory therapy with the purine analogues azathioprine or 6-mercaptopurine, methotrexate and/or anti-TNF-antibodies [1], [2], [3]. Azathioprine and 6-mercaptopurine are prodrugs, converted *in vivo* to active metabolites via a complex metabolism [4] (Figure S1). Two main metabolite groups are produced; the phosporylated thioguanine nucleotides (6-TGNs) which comprise thioguanosine mono-, di- and triphosphates, and methylated thioinosine phosphates measured as meTIMP [5]. Both metabolite groups contribute to the immunomodulatory effects in different ways [4], [6], [7], [8], [9]. Up to 30% of IBD-patients discontinue thiopurine therapy due to adverse events or refractoriness [1], [10], [11]. A cut off at >230–260 pmol 6-TGN/8×10^8^ red blood cells (RBC) has been proposed as a lower limit for clinical efficacy [12], but controversy exists regarding its utility, since an important overlap exists between patients in remission and those with active disease. High concentration of the other major metabolite, meTIMP, has mainly been associated with hepatotoxicity [13] and myelotoxicity [11]. Thus, a high meTIMP/6-TGN concentration ratio indicates an increased risk of both therapy failure and adverse events [14], [15], [16].

The xanthine oxidase inhibitor allopurinol in combination with a reduced dose of thiopurine (∼25–33% of original dose) may be considered in patients with this unfavourable metabolite profile. The combination therapy switches the metabolism towards enhanced 6-TGN production and has been shown safe and effective in IBD [15], [17]. It also reduces glucocorticosteroid requirements and hepatic as well as nonhepatic side effects [15], [17], [18]. The use of 5-ASA has also been suggested as an alternative to manipulate the metabolite profile by inhibition of TPMT [19], [20], [21]. However, the effect of 5-ASA on 6-TGN *in vivo* varies between studies [11], [13], [20], [22], [23], [24], [25], [26] and it does not seem to change the concentration of meTIMP [23], [24]. The use of 5-ASA to modulate the metabolite profile has not been implemented in clinical practice in a similar way as allopurinol.

The underlying mechanism to why a proportion of patients preferentially metabolize AZA and 6-MP to meTIMP is currently unknown. Interindividual variation in metabolite profiles and drug response may be explained by differences in activities of drug-metabolizing enzymes, and their correlation with corresponding transcript or protein levels. A well-known cause of adverse reactions to thiopurines is reduced or absent thiopurine S-methyltransferase (TPMT) activity, where patients with low TPMT activity accumulate myelotoxic concentrations of 6-TGNs if treated with standard doses [27]. However, not all interindividual differences in thiopurine metabolism and response can be attributed to variations in TPMT [14], [15], [28], [29], [30], since a large number of enzymes are involved.

Blockage of inosine 5′-monophosphate dehydrogenase (IMPDH) activity could, based on its position in the metabolic pathway of thiopurines (Figure S1), restrict the formation of 6-TGNs and therefore explain a high meTIMP/6-TGN concentration ratio. Indeed, the IMPDH activity was inversely correlated with the concentration of meTIMP in our previous work. However, no correlation with the concentration of 6-TGN was observed [25], [31].

Inosine triphosphatase (ITPase) is involved in a metabolic loop where 6-TIMP is reconverted from 6-thioinosine triphosphate (Figure S1). ITPase deficiency may contribute to the metabolite profile by increasing the concentration of methylated metabolites (methyl thioinosine triphosphate) [32]. Furthermore, most nucleoside analogues enter and exit cells via nucleoside transporters [33]. Impaired function, as well as up- or down-regulation of transport proteins with different specificities for thiopurine metabolites probably affect the metabolite profiles, as may variations in activities of intracellular nucleotidases and kinases.

The aim of this study was to explore the molecular basis of differences in metabolite profiles. We performed a whole genome expression analysis in blood samples from thiopurine treated patients with IBD and related the findings to metabolite concentrations and clinical patient characteristics.

## Materials and Methods

### Ethics Statement

The study was approved by the Ethics Committee at Linköping University, Sweden, dnr 03–260 and M58-06. Written informed consent was obtained from all patients.

### Patients

An explorative cohort of IBD-patients (n = 21) with normal TPMT activity (>8.9 U/mL pRBC; units per mL packed RBC) was selected based on differences in their metabolite profiles. We defined a high metabolite concentration ratio as meTIMP/6-TGN >20 based on metabolite determinations in our laboratory and on studies of deviant metabolism by others [14], [15], [34]. In our experience, this ratio corresponds to the 75^th^ percentile of the meTIMP/6-TGN concentration ratios (median 12) amongst 1220 patients with IBD on thiopurine therapy and TPMT activity in the normal range. Ten patients with meTIMP/6-TGN concentration ratios >20 (R20) were included in a microarray analysis, as were four patients with metabolite ratios corresponding to the median metabolite ratio (range 10.0–14.0, Median) and seven patients displaying a profile with a metabolite ratio ≤4 and 6-TGN ≥100 pmol/8×10^8^ RBC (R4). The cut-off ≥100 pmol 6-TGN/8×10^8^ RBC was employed to ensure acceptable analytical precision.

All patients had been on long term medication with an unchanged thiopurine dose for at least 2 weeks prior to blood sampling. Metabolite profiles were stable as judged from historical records, with at least two observations of the same kind available in 19/21 patients. Patients who had received blood transfusion within 4 months were not included**.** Disease activity (score ≥5 in active disease) was noted based on the Walmsley index for UC (n = 10) and the Harvey-Bradshaw index for CD (n = 10) and patient characteristics were noted (Table S1).

The data from the microarray analysis was further validated by reverse transcription quantitative PCR (RT qPCR) in an expanded patient cohort (n = 54), including the initial study population with the exception of one sample with insufficient amount of RNA (Table S2).

### Determination of TPMT Activity, and Thiopurine Metabolite Concentrations

TPMT activity, 6-TGN and meTIMP concentrations were measured in RBC [35], [36]. The interassay coefficient of variation in the TPMT assay was 8% at 12 U/mL pRBC. The interassay coefficients of variation at 72.5 and 785.2 pmol 6-TGN/8×10^8^ RBC were 13% and 11%, respectively. The interassay coefficients of variation at 1 624 and 17 524 pmol meTIMP/8×10^8^ RBC were 21% and 26%, respectively.

### Isolation of RNA from Peripheral Blood Samples

Blood samples were collected in PreAnalytiX Paxgene™ blood RNA tubes (Becton Dickinson, Franklin Lakes, NJ). RNA was isolated using the PreAnalytiX Paxgene™ blood RNA kit (Qiagen, Hilden, Germany) on the day of sampling, according to the manufacturers instructions. RNA concentration was assessed with Nanodrop® ND-1000 spectrophotometer (Nanodrop Technologies, Wilmington, DE) and RNA integrity with 2100 Bioanalyzer (Agilent technologies, Santa Clara, CA).

### Microarray Analysis

RNA samples were treated with the SnX™ globin depletion reagent [37] and analysed by AROS Applied Biotechnology (Aarhus, Denmark) with the Affymetrix GeneChip Human Genome U133 Plus 2.0 array (Affymetrix, Santa Clara, CA), representing the entire human genome with more than 38 500 well characterized genes.

### RT qPCR

Real-time PCR was performed with the FAST 7500 real-time PCR system and reagents from Applied Biosystems (Foster City, CA) with 5–10 ng cDNA per reaction in a final volume of 10 µL. Thirty-two potential reference genes (TaqMan® Express Human Endogenous Control Fast Plate, Applied Biosystems) were evaluated for low sample-to-sample variation using the Normfinder algorithm [38] and cDNA from six patients. The mRNA expression [C_T_ (threshold cycle)] of each target gene (Table S3) was normalized against the expression level of the selected reference genes (*GUSB*, *YWHAZ*, and *MRPL19*) with Genex Professional software version 4.3.8 (MultiD Analysis AB, Göteborg, Sweden) to obtain a delta-C_T_ (dC_T_). The relative expression was determined for each gene in relation to the sample with the lowest expression (highest C_T_).

By exploring the Pharmacogenomics knowledge base (http://www.pharmgkb.org/), the KEGG pathway of purine metabolism (http://www.genome.jp/kegg/pathway.html#nucleotide) and the thiopurine literature, 30 genes with a proven or potential relationship to the mechanisms, metabolism or transmembrane transport of purine/thiopurines were selected and analysed separately by RT qPCR (Table S3).

Probe set annotations and the corresponding TaqMan assays were obtained through Affymetrix NetAffx (http://www.affymetrix.com/analysis/index.affx) and Applied Biosystems UMapIt (http://www4.appliedbiosystems.com/tools/umapit/) (Table S3).

### Data Analysis

#### Analysis of microarray data

The image files (cel format) were imported to the GeneSpring GX 11 software (Agilent Technologies, Santa Clara, CA). Data was background corrected and normalized by the robust multiarray analysis algorithm [39].

In order to identify new candidate genes differently expressed over metabolite profiles, low-intensity signals were removed by a stringent filtering, retaining all data with a signal intensity greater than the 75^th^ percentile of all intensities. Thereafter, only intensities above the 20^th^ percentile in 100% of at least 1 of the 3 metabolite profiles were retained, leaving 7325 probe sets for further analysis. Genes differently expressed between metabolite profiles were identified by analysis of variance (ANOVA) without correction for multiple testing and with the Student-Newman-Keuls post hoc test at a level of statistical significance (*P*-value) of 0.001.

The 7325 probe sets were further included in a Spearman rank order correlation analysis against the individual dose-normalized metabolite concentrations and the meTIMP/6-TGN concentration ratio. The results were considered statistically significant if *P*<0.001.

#### Pathway analyses

In order to detect differences between metabolite profiles, gene set enrichment analysis (GSEA) was performed on the normalized data set, using the Broad Institute GSEA software, version 3.0 [40], [41]. Patients with meTIMP/6-TGN concentration ratio >20 were compared with patients with ratios ≤4 using the C2 molecular signatures database (MSigDB) of pathways of the Kyoto Encyclopedia of Genes and Genomes (KEGG), containing 186 gene set (414 pathways).

The interactions between gene products identified in this study and genes present at susceptibility loci identified for CD, UC and IBD [42] were evaluated with the Search Tool for the Retrieval of Interacting Genes/Proteins database, STRING, version 8.3 [43]. All prioritized candidate susceptibility genes were extracted from Table S2 of Jostins *et al.* [42], whereas for susceptibility loci with no prioritized genes, all genes were extracted. Candidate susceptibility genes with erroneous interactions with genes identified in this work were manually removed from the network.

#### Statistics

Dose-normalized metabolite concentrations (pmol metabolite per mg azathioprine) were used when investigating relationships between gene expression and metabolite concentrations. 6-mercaptopurine doses were converted to azathioprine doses, assuming a conversion factor of 2.08 [44].

Correlations between variables were evaluated using the Spearman rank order correlation coefficient, R_s_. Median (range) values are given. For group comparisons of continuous variables, the Mann-Whitney U-test, or the Kruskal Wallis test were used. For categorical variables, Fisheŕs exact test was used. Two-sided testing was used and considered statistically significant if *P*<0.05. Multiple linear regression analyses, using backward stepwise removal or inclusion of variables, were applied to assess the relationship between log transformed dose-normalized metabolite concentrations or meTIMP/6-TGN concentration ratios as dependent variables and gene expression levels expressed as dC_T_ values and the use of concomitant drugs as independent variables. A *P* to enter of 0.15 and a *P* to exit the models of 0.15 was adopted. Log transformation was necessary to normalise the distribution of the dependent variables.

Statistical analyses were performed using Statistica version 9.1 (StatSoft Inc, Tulsa, OK).

## Results

### Genes Identified by Microarray Screening - ANOVA

Four genes that were differently expressed over metabolite profiles were identified in the explorative patient cohort using the stringent filtering of microarray data (*P*<0.001); *FAM46A*, *SLX1A*, *TGOLN2*, *UBE2A* and included in the analyses with RT qPCR (Table S3).

### Genes Identified by Microarray Screening – Spearman Rank Order Correlation Analyses with Metabolite Concentrations

Among the significant genes identified by means of Spearman rank order correlation analyses of the 7325 probe sets, the top five or six genes with the most significant correlations with the individual metabolite concentrations, or the meTIMP/6-TGN concentration ratio, were selected for further evaluation.

The expression levels of *HVCN1*, *TOX4*, *SMAP2*, *DEF8*, and *PLCB2* were positively correlated with the concentration of 6-TGN (R_s_ 0.68–0.75; *P*≤0.0007). The expression level of *HVCN1* and *PLCB2* also correlated inversely with the meTIMP/6-TGN concentration ratio (R_s_−0.69–−0.67; *P*≤0.0008) (Table S4).

*UBE2A*, *FAM156A*, *CD1D*, *TUSC2*, and *GNB4* were inversely correlated with the concentration of meTIMP (R_s_−0.74–−0.72; *P*≤0.0002), whereas *MAP3K1* (probe id 243030_at, chromosomal alignment area associated with *MAP3K1*) correlated positively with this metabolite (R_s_ = 0.72; *P* = 0.0002). The expression levels of the genes associated with meTIMP also correlated with the meTIMP/6-TGN concentration ratio (*P*≤0.0009) (Table S4).

Additionally, the expression levels of *FAR1*, *LAP3* and *CTSS* were inversely associated with the meTIMP/6-TGN concentration ratio (R_s_ = 0.74, *P* = 0.0001).

In total, fourteen candidate genes were identified by Spearman rank correlation analyses, one of which overlapped with the four genes identified by ANOVA (*UBE2A*). No genes with known association with the metabolic pathways of thiopurine drugs or purines were identified in the microarray screen (employing a p-value <0.001).

### RT qPCR Data *vs.* Microarray Data – General Screening

**Figure 1:**
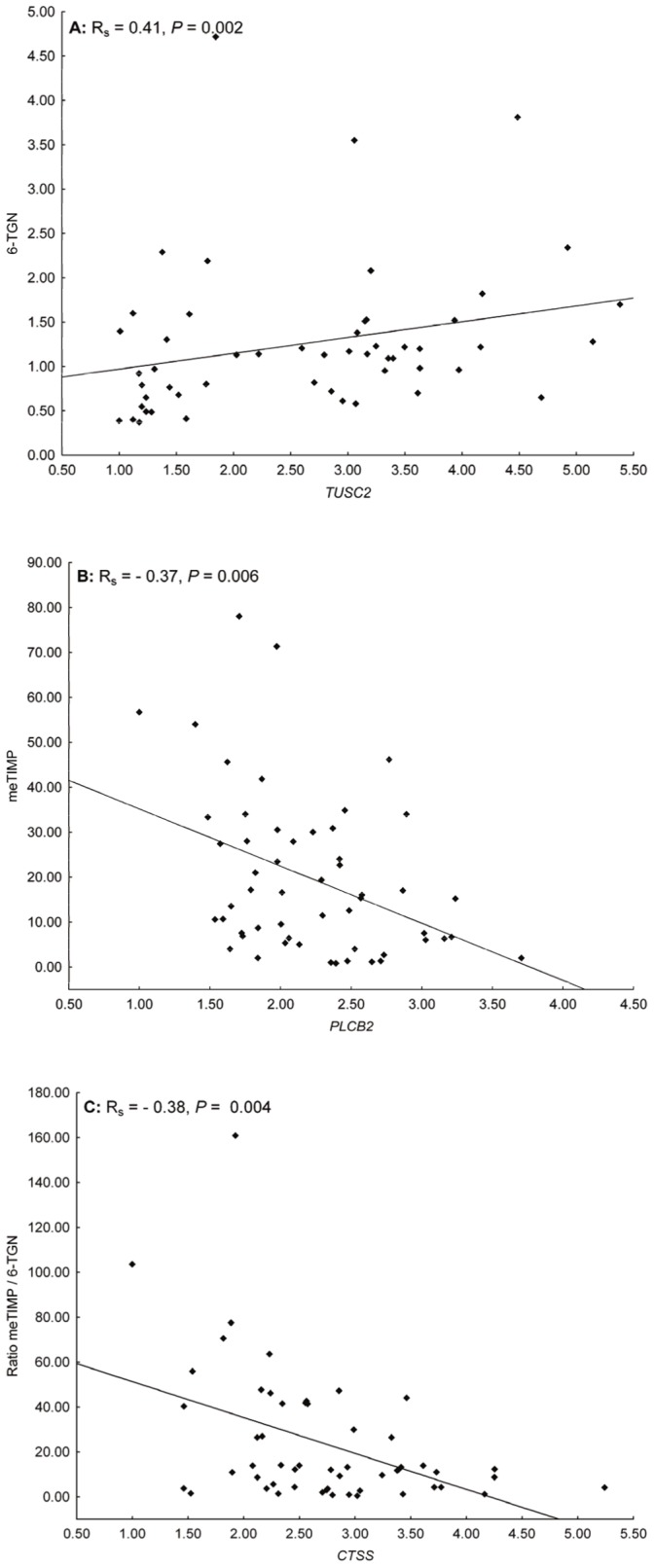
Scatterplots of genes identified by means of microarray screening (RT qPCR data). Scatterplots illustrating the co-variation between the relative gene expression levels of the genes with the most significant correlation coefficients with the concentration of A) 6-TGN, B) meTIMP or C) the meTIMP/6-TGN concentration ratio.

Altogether, 17 genes, identified by microarray screening, were taken to RT qPCR in the expanded patient cohort (n = 54) (Table S3). The relative gene expression levels of nine genes, correlated with the concentration of meTIMP, 6-TGN or the meTIMP/6-TGN concentration ratio (Table 1, Table S5, and exemplified in Figure 1).

**Table 1:** Spearman rank correlations (RT qPCR data *vs.* metabolite data)^a^, in the expanded patient cohort, of genes identified by microarray screening.

	6-TGN			meTIMP			Ratio meTIMP/6-TGN		
Gene	R_s_	*P*	Array^b^	R_s_	*P*	Array^b^	R_s_	*P*	Array^b^
*CD1D*	**0.27**	**0.05**		−0.26	0.06	N	−**0.33**	**0.02**	N
*CTSS*	0.16	0.26		−**0.39**	**0.003**		−**0.38**	**0.004**	N
*DEF8*	**0.32**	**0.02**	P	−0.17	0.24		−**0.27**	**0.05**	
*FAM156A*	0.18	0.18		−**0.33**	**0.01**	N	−**0.34**	**0.01**	N
*GNB4*	0.17	0.22		−**0.33**	**0.01**	N	−**0.37**	**0.006**	N
*HVCN1*	**0.32**	**0.02**	P	−0.09	0.51		−0.20	0.16	N
*LAP3*	0.24	0.08		−0.22	0.10		−**0.28**	**0.04**	N
*PLCB2*	0.19	0.17	P	−**0.37**	**0.006**		−**0.37**	**0.006**	N
*TUSC2*	**0.41**	**0.002**		−0.16	0.24	N	−**0.31**	**0.02**	N

a Genes that showed a significant correlation (*P*<0.05) between relative gene expression levels (RT qPCR data) and the concentration of 6-TGN, meTIMP or the meTIMP/6-TGN concentration ratio in the expanded patient cohort (n = 54).

b Significant (*P*<0.001) positive (P) or negative (N) correlations observed in the microarray data of the explorative patient cohort (n = 21) are indicated.

Four genes (*CD1D*, *DEF8*, *HVCN1*, and *TUSC2*) correlated positively with 6-TGN and all except *HVCN1* correlated negatively with the meTIMP/6-TGN concentration ratio. In the microarray data, *DEF8* and *HVCN1* displayed a significant positive correlation with 6-TGN, whereas the other two genes displayed a significant negative correlation with the meTIMP/6-TGN concentration ratio.

*CTSS*, *FAM156A*, *GNB4*, and *PLCB2* were negatively correlated with both the concentration of meTIMP and the meTIMP/6-TGN concentration ratio. In the microarray data, *FAM156A* and *GNB4* displayed a significant inverse correlation with meTIMP, whereas the other two genes displayed a significant inverse correlation with the meTIMP/6-TGN concentration ratio.

*LAP3* correlated negatively only with the meTIMP/6-TGN concentration ratio, both in the expanded cohort and in the microarray screen.

Substantial interindividual variation in the gene expression levels was observed, with a considerable overlap in expression levels between the three metabolite profiles (Table 2). Nevertheless, the gene expression levels of *CD1D*, *CTSS*, *FAM156A*, *GNB4*, and *PLCB2* were lower in patients with meTIMP/6-TGN concentration ratios >20 as compared with those with a median metabolite ratio (Median). Of these five differences, four were also noticed when comparing patients with high metabolite ratios (>20) with those with low metabolite ratios (≤4) (Table 2).

**Table 2:** Relative gene expression levels (RT qPCR data) of genes identified by microarray screening *vs.* metabolite profiles.^a.^

	All (n = 54)		R20 (n = 19)		Median (n = 17)		R4 (n = 18)		R20 *vs*. Median	R20 *vs*. R4	R4 *vs.* Median
Gene	Median	Range	Median	Range	Median	Range	Median	Range	*P*	*P*	*P*
*CD1D*	3.38	1.00–9.35	2.41	1.00–9.35	4.43	1.99–7.89	3.83	1.87–8.30	**0.005**	**0.03**	0.48
*CTSS*	2.64	1.00–5.24	2.23	1.00–3.46	2.93	1.90–4.25	2.78	1.46–5.24	**0.006**	**0.02**	0.66
*DEF8*	2.29	1.00–3.77	1.78	1.02–3.77	2.64	1.00–3.58	2.30	1.58–3.55	0.07	0.07	0.44
*FAM156A*	2.05	1.00–4.54	1.57	1.00–3.25	2.22	1.16–4.54	2.15	1.16–4.09	**0.04**	**0.02**	0.71
*GNB4*	2.03	1.00–3.60	1.70	1.00–2.73	2.31	1.33–3.61	2.11	1.20–3.26	**0.02**	0.06	0.94
*HVCN1*	2.23	1.00–3.63	1.88	1.01–3.56	2.40	1.09–3.63	2.25	1.00–3.22	0.07	0.33	0.37
*LAP3*	3.12	1.00–10.12	2.80	1.00–10.12	3.37	2.19–9.90	3.21	2.27–6.50	0.13	0.12	0.81
*PLCB2*	2.11	1.00–3.71	1.87	1.00–2.89	2.42	1.53–3.24	2.37	1.59–3.71	**0.01**	**0.02**	0.96
*TUSC2*	2.83	1.00–5.38	1.59	1.00–5.14	3.07	1.01–5.38	3.16	1.12–4.49	0.09	**0.04**	0.48

a Reverse transcription qPCR data, in the expanded patient cohort (n = 54), of genes showing significant results either in a Spearman rank order correlation analysis or in a Mann-Whitney U-test. Significant group differences are indicated in bold. Abbreviations: R20; meTIMP/6-TGN concentration ratio >20, Median; median metabolizer, R4; meTIMP/6-TGN concentration ratio ≤5.7.

### Genes Related to the Metabolic Pathway of Thiopurines

**Figure 2:**
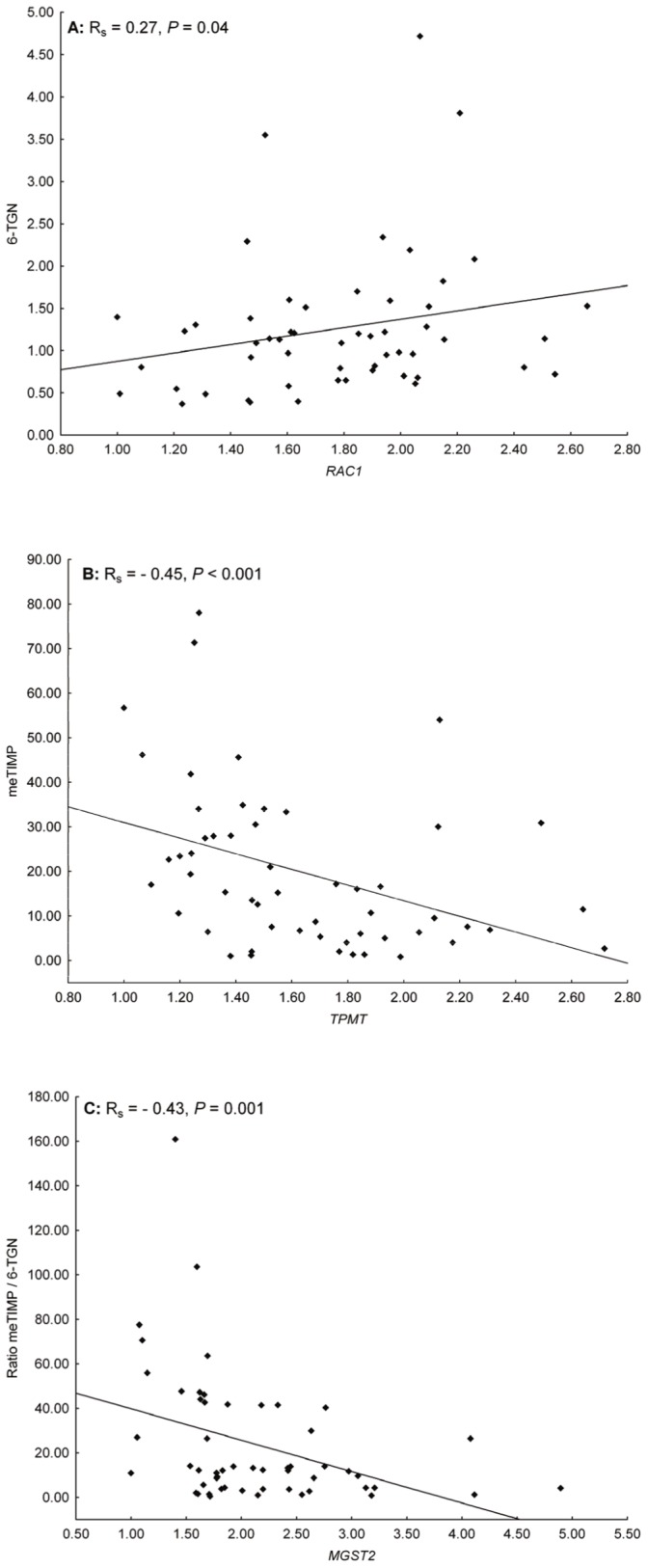
Scatterplots of genes selected for a proven or potential relationship to thiopurines (RT qPCR data). Scatterplots illustrating the co-variation between the relative gene expression levels of the genes with the most significant correlation coefficients with the concentration of A) 6-TGN, B) meTIMP or C) the meTIMP/6-TGN concentration ratio.

Thirty genes potentially associated with the mechanism, metabolism or transmembrane transport of thiopurines or purines, were analysed with RT qPCR (Table S3). When the relative gene expression levels were evaluated, nine genes correlated with the concentration of meTIMP, 6-TGN and/or the meTIMP/6-TGN concentration ratio (Table 3, Table S6, and exemplified in Figure 2).

**Table 3:** Spearman rank correlations (RT qPCR data *vs.* metabolite data)^a^, in the expanded patient cohort, of purine/thiopurine related genes.^b^

	6-TGN		meTIMP		Ratio meTIMP/6-TGN	
Gene	R_s_	*P*	R_s_	*P*	R_s_	*P*
*ENTPD1*	−0.10	0.49	−**0.27**	**0.05**	−0.21	0.12
*HPRT1*	−**0.28**	**0.04**	0.06	0.68	0.13	0.35
*IMPDH2*	−0.11	0.45	**0.29**	**0.03**	**0.30**	**0.03**
*MGST2*	0.20	0.15	−**0.41**	**0.002**	−**0.43**	**0.001**
*NME6*	0.24	0.08	−**0.27**	**0.05**	−**0.35**	**0.01**
*NT5E*	−0.09	0.51	**0.42**	**0.002**	**0.42**	**0.002**
*RAC1*	**0.27**	**0.04**	−0.24	0.08	−**0.37**	**0.006**
*SLC29A2*	0.02	0.89	**0.34**	**0.01**	**0.29**	**0.03**
*TPMT*	−0.12	0.38	−**0.45**	**<0.001**	−**0.34**	**0.01**

a Genes that showed a significant correlation (*P*<0.05) between relative gene expression levels (RT qPCR data) and the concentration of 6-TGN, meTIMP or the meTIMP/6-TGN concentration ratio in the expanded patient cohort (n = 54).

b None of the genes selected by means of their potential relationship with the mechanism, metabolism or transport of purines/thiopurines fulfilled the established significance level (*P*<0.001) in the microarray data.

A positive correlation was observed between the gene expression level *RAC1* and the concentration of 6-TGN, whereas *HPRT1* correlated negatively with this metabolite (both *P* = 0.04).

The expression levels of *ENTPD1, MGST2*, *NME6*, and *TPMT* were inversely correlated with the concentration of meTIMP (*P*≤0.046), and *IMPDH2*, *NT5E*, and *SLC29A2* were positively correlated with this metabolite (*P*≤0.03).

The expression levels of *MGST2*, *NME6*, *RAC1*, and *TPMT* were negatively correlated with the meTIMP/6-TGN concentration ratio (*P*≤0.01), and *IMPDH2*, *NT5E*, and *SLC29A2* were positively correlated with the ratio (*P*≤0.03).

Two genes, *XDH* and *NT5C1B*, were not expressed in peripheral blood as judged by the RT qPCR data.

Patients with meTIMP/6-TGN ratios >20 showed lower expression levels of *MGST2, TPMT* and *NME6,* but higher expression levels of *IMPDH2* and *NT5E* than patients with metabolite ratios ≤4. However, a large overlap in gene expression levels over the three metabolite profiles was observed (Table 4).

**Table 4:** Relative gene expression levels (RT qPCR data) of purine/thiopurine related genes *vs.* metabolite profiles.^a^

	All (n = 54)		R20 (n = 19)		Median (n = 17)		R4 (n = 18)		R20 *vs.* Median	R20 *vs.* R4	R4 *vs.* Median
Gene	Median	Range	Median	Range	Median	Range	Median	Range	*P*	*P*	*P*
*ENTPD1*	1.70	1.00–4.85	1.61	1.00–4.85	1.83	1.15–3.61	1.77	1.18–4.25	0.57	0.26	0.52
*HPRT1*	1.42	1.00–1.97	1.46	1.00–1.93	1.36	1.09–1.97	1.42	1.25–1.64	0.15	0.31	0.59
*IMPDH2*	1.86	1.00–2.95	1.92	1.47–2.95	1.89	1.12–2.25	1.75	1.00–2.19	0.57	**0.05**	0.22
*MGST2*	1.86	1.00–4.90	1.66	1.06–4.08	2.11	1.00–3.06	2.17	1.59–4.90	**0.04**	**0.01**	0.46
*NME6*	1.58	1.00–2.35	1.38	1. 5–2.05	1.71	1.00–2.35	1.62	1.19–2.19	0.06	**0.03**	0.73
*NT5E*	9.25	1.00–32.14	11.28	2.12–32.14	8.88	2.30–18.74	6.93	1.00–26.94	0.17	**0.05**	0.24
*RAC1*	1.80	1.00–2.66	1.60	1.01–2.44	1.85	1.00–2.54	1.97	1.28–2.66	0.26	0.05	0.42
*SLC29A2*	2.22	1.00–3.68	2.37	1.42–3.39	2.16	1.14–3.68	1.97	1.00–3.43	0.29	0.10	0.55
*TPMT*	1.53	1.00–2.72	1.41	1.00–2.49	1.52	1.10–2.64	1.78	1.30–2.72	0.53	**0.02**	0.23

a Reverse transcription qPCR data, in the expanded patient cohort (n = 54), of genes showing significant results either in a Spearman rank order correlation analysis or in a Mann-Whitney U-test. Significant group differences are indicated in bold. Abbreviations: R20; meTIMP/6-TGN concentration ratio >20, Median; median metabolizer, R4; meTIMP/6-TGN concentration ratio ≤5.7.

The TPMT activity in RBC did not correlate with the mRNA expression of *TPMT* (R_s_ = 0.10, *P* = 0.45).

### Pathway Analysis; STRING and GSEA

The GSEA showed no significant enrichment of genes of any pathway in any phenotype (different metabolite profiles) when a false discovery rate of 0.25 and 1000 permutations of each phenotype was applied (data not shown). However, *RAC1*, *PLCB2* and *GNB4* overlapped with the KEGG chemokine signaling pathway (*P* = 4.6×10^−5^) and *RAC1* and *PLCB2* with the KEGG Wnt signaling pathway (*P* = 0.002).

**Figure 3:**
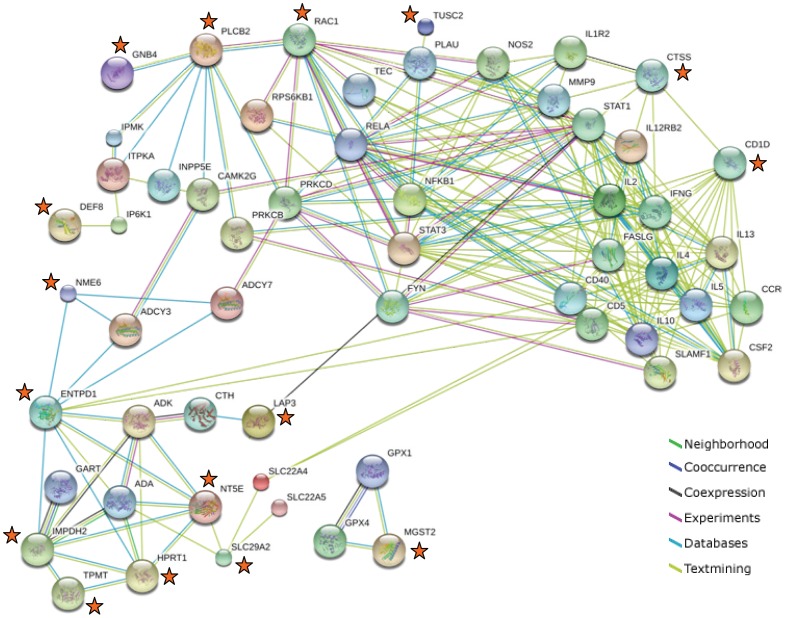
Protein-protein network analysis; STRING database. Interactions between candidate IBD susceptibility genes and genes identified as correlated with meTIMP, 6-TGN and/or the meTIMP/6-TGN concentration ratio (marked with an orange star).

Sixteen out of 18 genes (89%) which correlated significantly with meTIMP and/or 6-TGN or the meTIMP/6-TGN concentration ratio in the expanded patient cohort (n = 54) interacted in networks with 41 genes present at 6, 4 or 21 susceptibility loci identified for CD, UC or IBD [42], respectively, as judged by the STRING analysis (Figure 3; *FAM156A* and *HVCN1* were not present in any network). All the network-identified candidate susceptibility genes belonged to genes prioritized by Jostins *et al*. [42] with the exception of four genes at two susceptibility loci without prioritized genes (*CTH*, *ADK*, *CAMK2G* and *PLAU*). Genes selected for a potential association with the metabolism (*IMPDH2*, *HPRT1*, *TPMT*, *NT5E*, *ENTPD1* and *NME6*) or transmembrane transport (*SLC29A2*) of thiopurines or purines connected with candidate susceptibility genes linked to the KEGG pathway of purine metabolism, except *MGST2* which linked to candidate susceptibility genes involved in the KEGG pathway of glutathione metabolism (Figure 3, and data not shown). Genes identified by microarray screening were linked to the following KEGG pathways through interactions with candidate susceptibility genes: an inter-pathway connection between ‘cysteine and methionine metabolism’ and ‘glutathione metabolism’ (*LAP3*), inositol phosphate metabolism and signaling through phosphatidylinositol, calcium, chemokine or Wnt (*PLCB2*), chemokine and Wnt signaling (*RAC1*), chemokine signaling (*GNB4*) (Figure 3, and data not shown). For four of the genes (*CD1D*, *CTSS*, *TUSC2*, and *DEF8*), the STRING analysis identified interactions solely based on textmining and indicated involvement in functioning of invariant natural killer T cells, T cells and B cells, cytokine and chemokine signaling, anti-proliferative and apoptotic effects, and inositol phosphate metabolism (Figure 3, and data not shown).

### Active Disease *vs.* Remission

The gene expression levels of *CTSS*, *DEF8*, *HVCN1*, *NME6*, and *PLCB2* were higher in remission than in active disease (*P*≤0.04), whereas a decreased expression of *IMPDH2* was associated with remission (*P* = 0.008). Eight of 9 patients with active disease had CD. Considering CD patients only, the results were essentially the same with inverse relationships between the gene expression levels of *CTSS* (R_s_ = −0.39), *DEF8* (R_s_ = −0.53), *HVCN1* (R_s_ = −0.44), *NME6* (R_s_ = −0.43), *PLCB* (R_s_ = −0.47) and the Harvey-Bradshaw index (*P*≤0.04).

The measured concentrations of meTIMP and 6-TGN were no different in patients in remission (n = 43) compared with those with active disease (n = 9, *P*≥0.13).

### Regression Analyses

All genes that displayed an individual correlation with one of the metabolites or the meTIMP/6-TGN concentration ratio in the expanded patient cohort, were, together with concomitant therapy with 5-ASA or glucocorticosteroids (distribution between metabolite profiles, *P* = 0.11 and 0.006, respectively) assessed using multiple linear regression analyses.

The regression models obtained explained at most 46, 35 and 27% of the variation in the meTIMP/6-TGN concentration ratio, the concentration of meTIMP and 6-TGN, respectively (Table 5, 6, 7).

**Table 5:** A multiple linear regression model for the meTIMP/6-TGN concentration ratio.^a^

Parameter		Estimate	95% CI	*P*
Intercept		0.44	−3.32–4.20	0.82
*CD1D*		−1.04	−1.81–−0.27	0.01
*TUSC2*		1.02	0.39–1.64	0.002
*PLCB2*		1.47	0.42–2.52	0.007
*NT5E*		−0.29	−0.64–0.07	0.11
*TPMT*		1.31	0.28–2.34	0.01
Glucocorticosteroids	Yes	0.57	0.14–0.99	0.01

a The regression model is based on the expanded patient cohort (n = 54), using the natural logarithm of the meTIMP/6-TGN concentration ratio as dependent variable and the gene expression levels (dC_T_ values) of *CD1D*, *PLCB2*, *NT5E*, *TPMT*, *TUSC2*, and glucocorticosteroid therapy as independent variables. A higher dC_T_ value is associated with a lower gene expression level. *R^2^* = 0.46, *P* = 3×10^−5^.

**Table 6:** A multiple linear regression model for the concentration of meTIMP.^a^

Parameter		Estimate	95% CI	*P*
Intercept		0.60	−2.45–3.66	0.69
*PLCB2*		1.02	0.27–1.78	0.009
*TPMT*		0.97	0.12–1.81	0.03
5-ASA	Yes	−0.39	−0.77–−0.01	0.04
5-ASAxGlucocorticosteroids	Yes	−0.31	−0.68–0.06	0.10

a The regression model is based on the expanded patient cohort (n = 54), using the natural logarithm of the meTIMP concentration as dependent variable and the gene expression levels (dC_T_ values) of *PLCB2* and *TPMT* and glucocorticosteroid and 5-ASA therapy as independent variables. A higher dC_T_ value is associated with a lower gene expression level. *R^2^* = 0.35, *P* = 2×10^−4^.

**Table 7:** A multiple linear regression model for the concentration of 6-TGN.^a^

Parameter		Estimate	95% CI	*P*
Intercept		0.08	−0.16–0.31	0.52
*TUSC2*		−0.27	−0.46–−0.07	0.008
Glucocorticosteroids	Yes	−0.22	−0.41–−0.04	0.02
5-ASAxGlucocorticosteroids	Yes	−0.11	−0.25–0.03	0.14

a The regression model is based on the expanded patient cohort (n = 54), using the natural logarithm of the 6-TGN concentration as dependent variable and the gene expression level (dC_T_ value) of *TUSC2*, and glucocorticosteroid and 5-ASA therapy as independent variables. A higher dC_T_ value is associated with a lower gene expression level. *R^2^* = 0.27, *P* = 0.001.

The models were essentially the same using untransformed data (data not shown).

### 5-ASA and Glucocorticosteroid Therapy

The gene expression level of *TPMT* was higher among patients on 5-ASA therapy (n = 26) compared with those without 5-ASA (n = 28), [1.76 (range 1.27–2.72) *vs.* 1.42 (1.00–2.49), *P* = 0.008]. However, there was no difference in TPMT activity [11.9 U/mL pRBC (8.7–18.2) *vs.* 13.4 U/mL pRBC (9.4–15.8), *P* = 0.38] even though the concentration of meTIMP was lower in patients on 5-ASA compared with those without 5-ASA [1400 pmol/8×10^8^ RBC (100–11700) *vs.* 3150 pmol/8×10^8^ RBC (100–10700), *P* = 0.01]. The concentration of 6-TGN did not differ between the two groups [163.9 pmol/8×10^8^ RBC (71.8–710.7) *vs.* 147.9 pmol/8×10^8^ RBC (64.4–351.2), *P* = 0.97].

The gene expression levels of *CD1D*, *CTSS*, *DEF8*, *FAM156A*, *MGST2*, and *NME6* were lower in patients on glucocorticosteroid therapy (n = 10) *vs.* those who were not (n = 44, *P*≤0.02). However, there was a large overlap in gene expression levels between the two groups (data not shown).

## Discussion

An aberrant thiopurine metabolism with preferential formation of the methylated metabolites has been related to lesser efficacy and an increased risk of adverse events [11], [14], [15], [16]. At present it is unknown why up to 25% of patients display such a metabolite profile. In order to explore the molecular basis of differences in thiopurine metabolite concentrations we adopted a broad approach using whole genome expression analysis in blood samples from patients with distinct metabolite profiles.

Based on the microarray screening we selected seventeen candidate genes for further analysis using RT qPCR in the expanded patient cohort, and nine of these genes demonstrated significant correlations with meTIMP and/or 6-TGN concentrations. However, among the genes there was none with a known or suspected relationship to the purine/thiopurine metabolism, transport or drug effects. Furthermore, although large interindividual differences in gene expression levels were observed (Table 2 and Table 4), only small differences were noticed between the three metabolite profiles (R20, Median and R4). Thus, based on gene expression, we did not find a strong influence on the three distinct thiopurine metabolite profiles.

Thirty genes potentially associated with the mechanism, metabolism or transmembrane transport of thiopurines or purines were explored by means of RT qPCR in the expanded patient cohort (irrespective of the microarray result). The expression levels of nine genes correlated significantly with meTIMP and/or 6-TGN concentrations. All of these genes, except *RAC1*, putatively affect metabolism or transport of thiopurines or purines. *RAC1* encodes a small GTPase and is involved in the signaling from the T-cell receptor in activated CD4+ cells. Its downstream targets promote cellular survival. The 6-TGN metabolite 6-TGTP has the potential to induce apoptosis in these cells by blocking the Rac1 protein [7]. Rac1 also facilitates the interaction between antigen presenting cells and effector cells, a process disturbed by 6-TGTP [45].

Multiple regression analyses, using gene expression levels and concomitant therapy with 5-ASA or corticosteroids as predictors, explained at most 46% of the variation in the dependent metabolite variables. Four of the five genes included in the regression models had no established prior relationship with the metabolic scheme of thiopurines, although *NT5E* has been suggested to facilitate cellular uptake of thiopurine metabolites by extracellular de-phosphorylation [46]. Possibly, the thiopurine metabolite profile depends on several interacting genes, each with an individual small effect. However, it is also possible that the metabolite profile mainly reflects a post-transcriptional regulation. In clinical practise, thiopurine metabolites are measured in RBC, whereas gene expression levels were measured in the target cells of therapy. The use of RBC as a surrogate compartment for the target cells of therapy (the mononuclear cells) may obscure the ability to establish relationships between gene expression levels and metabolite concentrations.

No apparent relationships to purine/thiopurine metabolism were identified for the genes identified from the microarray screening. Therefore, gene expression levels were investigated in relation to disease activity. Five genes (*NME6* from the purine/thiopurine network, *PLCB2* of the *RAC1* network, and *HVCN1*, *CTSS*, and *DEF8*) correlated with the Harvey-Bradshaw index of Crohn’s disease activity. Sixteen genes out of the 18 genes that were verified using the expanded patient cohort as significantly correlated to thiopurine metabolite concentrations were connected to network of genes that originated from susceptibility loci for CD, UC or IBD [42]. Apart from interactions with candidate IBD susceptibility genes involved in purine metabolism, these interactions identified biological processes such as cytokine production and function of T cells, B cells and natural killer cells, corresponding to the most significantly enriched Gene Ontology terms identified by Jostins *et al.* in relation to candidate IBD susceptibility genes [42]. Additionally, inositol phosphate metabolism and signaling were identified as a potential target for the thiopurine drug mechanisms.

Apart from an involvement in thiopurine metabolite turnover, we conclude that the genes uncovered in this study to a large extent relate to drug targets and disease mechanisms of IBD.

## Supporting Information

Figure S1 **Schematic pathways of azathioprine (AZA) and 6-mercaptopurine (6-MP) metabolism.** GST, Glutathione transferase; GSH glutathione; XO, xanthine oxidase; AO, aldehyde oxidase; HGPRT, hypoxanthine guanine phosphoribosyltransferase; TPMT, thiopurine S-methyltransferase; SAM, S-adenosyl methionine; IMPDH, inosine 5′-monophosphate dehydrogenase; NAD, nicotine adenosine dinucleotide; ITPase, inosine triphosphatase; GMPS, guanosine monophosphate synthetase; GMP reductase, guanosine monophosphate reductase; GMP kinase, guanylate kinase; RNR, ribonucleotide reductase; NDPK, nucleotide diphosphate kinases; 6-TU, 6-thiouric acid; 6-TIMP, 6-thioinosine monophosphate; 6-TXMP, 6-thioxanthosine monophosphate; 6-TITP, 6-thioinosine triphosphate; meTIMP, methyl thioinosine monophosphate; 6-TGMP, 6-thioguanosine monophosphate; 6-TGDP, 6-thioguanosine diphosphate; 6-TGTP, 6-thioguanosine triphosphate; d, deoxy; 6-TGNs, 6-thioguanine nucleotides; PRPP, 5-phosphoribosyl-1-pyrophosphate; PRA, 5-phosphoribosylamine; AMP, adenosine monophosphate.
(PDF)

Table S1 **Characteristics of the exploratory patient cohort (n = 21).^a^**
(DOC)

Table S2 **Characteristics of the expanded patient cohort (n = 54).^a^**
(DOC)

Table S3 **Annotations of probe sets and genes and corresponding TaqMan® assays.**
(DOC)

Table S4 **Spearman rank correlations (microarray screening).^a^**
(DOC)

Table S5 **Spearman rank correlations (RT qPCR data** ***vs.*** **metabolite data)^a^ of genes identified by microarray screening.**
(DOC)

Table S6 **Spearman rank correlations (RT qPCR data** ***vs.*** **metabolite data)^a^ of genes with a proven or potential relation to purines/thiopurines.**
(DOC)
