# Supplementary figures and images for: Gene Expression and Thiopurine Metabolite Profiling in Inflammatory Bowel Disease – Novel Clues to Drug Targets and Disease Mechanisms?
Source: PLoS One. 2013 Feb 21;8(2):e56989. doi: 10.1371/journal.pone.0056989 (PMC3578787; doi:10.1371/journal.pone.0056989)

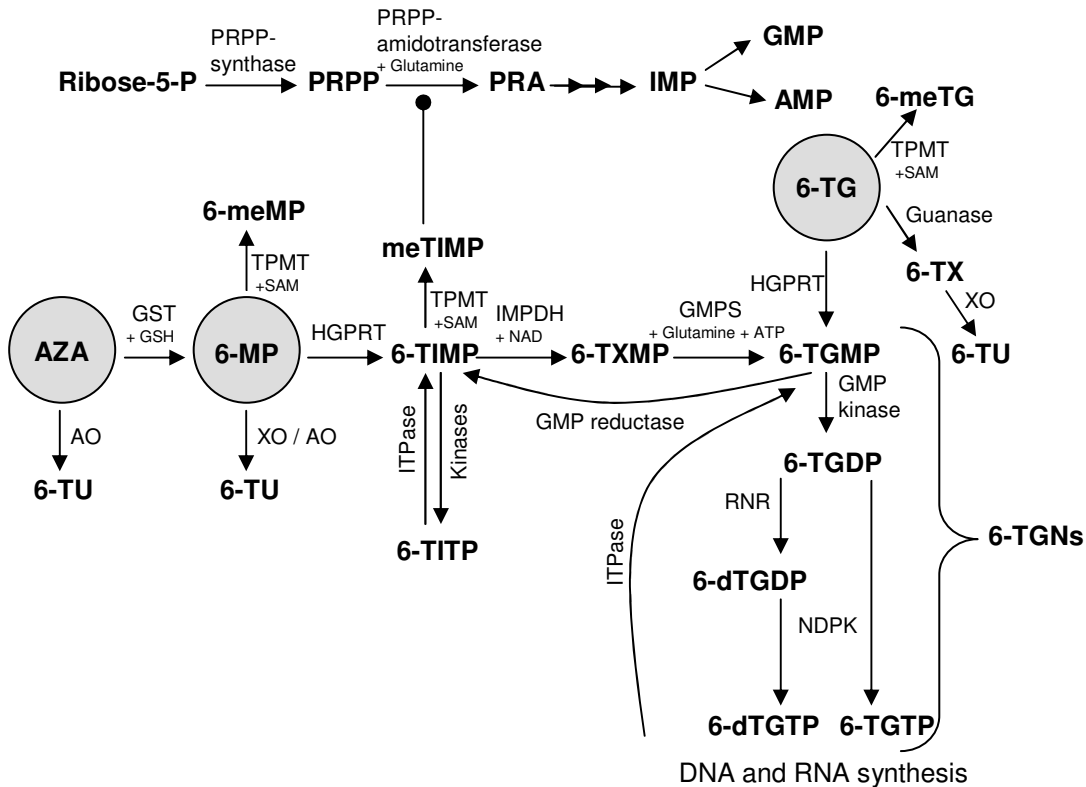

Supplement: Figure S1 — Schematic pathways of azathioprine (AZA) and 6-mercaptopurine (6-MP) metabolism. GST, Glutathione transferase; GSH glutathione; XO, xanthine oxidase; AO, aldehyde oxidase; HGPRT, hypoxanthine guanine phosphoribosyltransferase; TPMT, thiopurine S-methyltransferase; SAM, S-adenosyl methionine; IMPDH, inosine 5′-monophosphate dehydrogenase; NAD, nicotine adenosine dinucleotide; ITPase, inosine triphosphatase; GMPS, guanosine monophosphate synthetase; GMP reductase, guanosine monophosphate reductase; GMP kinase, guanylate kinase; RNR, ribonucleotide reductase; NDPK, nucleotide diphosphate kinases; 6-TU, 6-thiouric acid; 6-TIMP, 6-thioinosine monophosphate; 6-TXMP, 6-thioxanthosine monophosphate; 6-TITP, 6-thioinosine triphosphate; meTIMP, methyl thioinosine monophosphate; 6-TGMP, 6-thioguanosine monophosphate; 6-TGDP, 6-thioguanosine diphosphate; 6-TGTP, 6-thioguanosine triphosphate; d, deoxy; 6-TGNs, 6-thioguanine nucleotides; PRPP, 5-phosphoribosyl-1-pyrophosphate; PRA, 5-phosphoribosylamine; AMP, adenosine monophosphate. (PDF) [file pone.0056989.s001.pdf]
