# Supplementary material for: Gene Expression and Thiopurine Metabolite Profiling in Inflammatory Bowel Disease – Novel Clues to Drug Targets and Disease Mechanisms?
Source: PLoS One. 2013 Feb 21;8(2):e56989. doi: 10.1371/journal.pone.0056989 (PMC3578787; doi:10.1371/journal.pone.0056989)
Supplement: Table S1 — Characteristics of the exploratory patient cohort (n = 21).a (DOC) [file pone.0056989.s002.doc]

**Table S1.** Characteristics of the exploratory patient cohort (n = 21).^a^

|  | **All (n =21)** | **R20 (n = 10)** | **Median (n = 4)** | **R4 (n = 7)** | ***P*-value** |
| --- | --- | --- | --- | --- | --- |
| Disease (CD/UC/Other) | 10 / 10 / 1 | 6 / 3 / 1 | 2 / 2 | 2 / 5 | 0.32 |
| Gender (female/male) | 10 / 11 | 6 / 4 | 2 / 2 | 2 / 5 | 0.44 |
| Age (years) | 36 (23-80) | 45 (31-69) | 25 (24-47) | 31 (23-80) | 0.08 |
| Smoker (yes/no) | 4 / 17 | 3 / 7 | 0 / 4 | 1 / 6 | 0.40 |
| Remission/active disease^b^ | 18 / 2 | 7 / 2 | 4 / 0 | 7 / 0 | 0.26 |
| Corticosteroids (yes/no) | 6 / 15 | 5 / 5 | 0 / 4 | 1 / 6 | 0.19 |
| Mesalazine (yes/no) | 11 / 10 | 2 / 8 | 3 / 1 | 6 / 1 | 0.01 |
| Azathioprine/6-mercaptopurine | 18 / 3 | 8 / 2 | 3 / 1 | 7 / 0 | 0.40 |
| Azathioprine (mg/kg BW/day) | 2.2 (0.8-3.0) | 2.2 (1.9-3.0) | 2.6 (2.5-3.0) | 1.9 (0.8-2.9) | 0.10 |
| 6-mercaptopurine (mg/kg BW/day) | 0.7 (0.7-0.8) | 0.7 (0.7-0.8) | 0.7 | - | 1.0 |
| TPMT activity (U/mL pRBC) | 12.4 (10.4-18.2) | 12.7 (10.9-15.8) | 11.3 (10.4-18.2) | 12.4 (10.4-15.5) | 0.63 |
| 6-TGN (pmol/8x10^8^ RBC) | 140.5 (67.9-269.5) | 109.9 (67.9-151) | 197.9 (122.9-244.4) | 211.3 (97.4-269.5) | 0.006 |
| meTIMP (pmol/8x10^8^ RBC)^c^ | 2900 (0–11700) | 6650 (2900-11700) | 2290 (1700-2900) | 200 (0-900) | <0.001 |

^a^ Median (range) values are given. Abbreviations: R20; meTIMP/6-TGN concentration ratio >20, Median; median metabolizer, R4; meTIMP/6-TGN concentration ratio ≤4; 6-TGN; 6-thioguanine nucleotides, BW; body weight, CD; Crohn´s disease, meTIMP; methyl thioinosine monophosphate, TPMT; thiopurine S-methyltransferase, UC; ulcerative colitis.

^b^ Not applicable on one patient with other diagnosis (autoimmune hepatitis).

^c^ meTIMP: The lowest calibrator was 300 pmol/8x10^8^ RBC. If a result was reported as traces of meTIMP, it was set to a concentration of 200, and to 0 if reported as not detectable.
