# Supplementary material for: Gene Expression and Thiopurine Metabolite Profiling in Inflammatory Bowel Disease – Novel Clues to Drug Targets and Disease Mechanisms?
Source: PLoS One. 2013 Feb 21;8(2):e56989. doi: 10.1371/journal.pone.0056989 (PMC3578787; doi:10.1371/journal.pone.0056989)
Supplement: Table S2 — Characteristics of the expanded patient cohort (n = 54).a (DOC) [file pone.0056989.s003.doc]

**Table S2. Characteristics of the expanded patient cohort (n = 54).^a^**

|  | **All (n = 54)** | **R20 (n = 19)** | **Median (n = 17)** | **R4 (n = 18)** | ***P*-value** |
| --- | --- | --- | --- | --- | --- |
| Disease (CD/UC/Other) | 28 / 24 / 2 | 10 / 7 / 2 | 9 / 8 | 9 / 9 | 0.94 |
| Gender (female/male) | 28 / 26 | 13 / 6 | 9 / 8 | 6 / 12 | 0.11 |
| Age (years) | 36 (16-80) | 41 (18-69) | 30 (16-61) | 32 (18-80) | 0.36 |
| Smoker (yes/no) | 7 / 47 | 3 / 16 | 2 / 15 | 2 / 16 | 1.00 |
| Remission/active disease^b^ | 43 / 9 | 11 / 6 | 16 / 1 | 16 / 2 | 0.09 |
| Corticosteroids (yes/no) | 10 / 44 | 8 / 11 | 1 / 16 | 1 / 17 | 0.006 |
| Mesalazine (yes/no) | 26 / 28 | 6 / 13 | 8 / 9 | 12 / 6 | 0.11 |
| Azathioprine/6-mercaptopurine | 47 / 7 | 17 / 2 | 15 / 2 | 15 / 3 | 0.89 |
| Azathioprine (mg/kg BW/day) | 2.1 (0.8-3.4) | 2.2 (1.7-3.4) | 2.1 (1.7-3.0) | 2.0(0.8-2.7) | 0.16 |
| 6-mercaptopurine (mg/kg BW/day) | 0.7 (0.5-1.6) | 0.8 (0.7-0.8) | 1.1 (0.7-1.5) | 0.5 (0.5-1.6) | 0.70 |
| TPMT activity (U/mL pRBC)^c^ | 12.9 (8.7-18.2) | 12.1 (10.9-15.8) | 13.3 (9.4-18.2) | 13.1 (8.7-17.0) | 0.76 |
| 6-TGN (pmol/8x10^8^ RBC) | 160.8 (64.4-710.7) | 133.9 (64.4-192.5) | 182.4 (102.0-351.2) | 175.8 (97.4-710.7) | 0.003 |
| meTIMP (pmol/8x10^8^ RBC)^d^ | 2200 (100-11700) | 5400 (2900-11700) | 2100 (1100-3600) | 550 (100-2700) | <0.001 |

^a^ Median (range) values are given. Abbreviations: R20; meTIMP/6-TGN concentration ratio >20, Median; median metabolizer, R4; meTIMP/6-TGN concentration ratio ≤5.7; 6-TGN; 6-thioguanine nucleotides, BW; body weight, CD; Crohn´s disease, meTIMP; methyl thioinosine monophosphate, TPMT; thiopurine S-methyltransferase, UC; ulcerative colitis.

^b^ Not applicable on two patient with other diagnosis (autoimmune hepatitis).

^c^ One patient with TPMT activity below the cut of for normal TPMT activity (8.9 U/mL pRBC), but with wild type genotype.

^d^ meTIMP: The lowest calibrator was 300 pmol/8x10^8^ RBC. If a result was reported as traces of meTIMP, it was set to a concentration of 200, and to 100 if reported as not detectable (two cases).
