# Supplementary material for: Gene Expression and Thiopurine Metabolite Profiling in Inflammatory Bowel Disease – Novel Clues to Drug Targets and Disease Mechanisms?
Source: PLoS One. 2013 Feb 21;8(2):e56989. doi: 10.1371/journal.pone.0056989 (PMC3578787; doi:10.1371/journal.pone.0056989)
Supplement: Table S3 — Annotations of probe sets and genes and corresponding TaqMan® assays. (DOC) [file pone.0056989.s004.doc]

**Table S3.** Annotations of probe sets and genes and corresponding TaqMan® assays.

| **Probe ID** | **Gene symbol** | **TaqMan assay** | **Description** |
| --- | --- | --- | --- |
| **Reference genes** | | | |
|  | GUSB | Hs99999908_m1 | Beta-glucuronidase |
|  | YWHAZ | Hs00237047_m1 | Protein kinase C inhibitor protein 1 |
|  | MRPL19 | Hs00608519_m1 | 39S ribosomal protein L19 |
| **Genes identified by microarray screening - ANOVA** | | | |
| 221766_s_at | FAM46A | Hs00214159_m1 | Family with sequence similarity 46, member A |
| 218317_x_at | SLX1A | Hs02341353_g1 | SLX1 structure-specific endonuclease subunit homolog A, GIY-YIG domain-containing protein 1 |
| 1554608_at | TGOLN2 | Hs00197728_m1 | Trans-Golgi network integral membrane protein 2 precursor |
| 201899_s_at | UBE2A | Hs00163308_m1 | Ubiquitin-conjugating enzyme E2A |
| **Genes identified by microarray screening – Spearman rank correlation** | | | |
| 205789_at | CD1D | Hs00939888_m1 | Cd1d molecule |
| 232617_at | CTSS | Hs00175407_m1 | Cathepsin S |
| 225637_at | DEF8 | Hs00276462_m1 | Differentially expressed in FDCP 8 homolog |
| 223203_at | FAM156A | Hs00739497_s1 | Family with sequence similarity 156, member A |
| 224865_at | FAR1 | Hs00386153_m1 | Fatty acyl CoA reductase 1 |
| 225710_at | GNB4 | Hs01118085_m1 | Guanine nucleotide binding protein (G protein), beta polypeptide 4 |
| 226879_at | HVCN1 | Hs01032834_m1 | Hydrogen voltage-gated channel 1 |
| 217933_s_at | LAP3 | Hs00429769_m1 | Leucine aminopeptidase 3 |
| 243030_at | MAP3K1_custom | Custom made | Designed based on the chromosomal alignment area of the probe set |
| 204046_at | PLCB2 | Hs01080542_m1 | Phospholipase C, beta 2 |
| 244357_at | SMAP2 | Hs01077385_m1 | Small ArfGAP2, stromal membrane-associated GTPase-activating protein 2 |
| 201683_x_at | TOX4 | Hs00706258_s1 | TOX high mobility group box family member 4 |
| 203273_s_at | TUSC2 | Hs00200725_m1 | Tumor suppressor candidate 2 |
| 201899_s_at | UBE2A | Hs00163308_m1 | Ubiquitin-conjugating enzyme E2A |
| **Genes with a proven or potential relation to purines/thiopurines** | | | |
| 203196_at | ABCC4 | Hs00988717_m1 | Multidrug resistance-associated protein 4, ATP-binding cassette, sub-family C, member 4 |
| 1558460_at | ABCC5 | Hs00981089_m1 | Multidrug resistance-associated protein 5, ATP-binding cassette, sub-family C, member 5 |
| 207016_s_at | ALDH1A2 | Hs0018054_m1 | Aldehyde dehydrogenase 1 family, member A2 |
| 1554327_a_at | CANT1 | Hs00386220_m1 | Soluble calcium-activated nucleotidase 1 |
| 203716_s_at | DPP4 | Hs00175210_m1 | Dipeptidyl-peptidase 4 |
| 209473_at | ENTPD1 | Hs00969559_m1 | Ectonucleoside triphosphate diphosphohydrolase 1 |
| 205757_at | ENTPD5 | Hs04176260_g1 | Ectonucleoside triphosphate diphosphohydrolase 5 |
| 204187_at | GMPR1 | Hs00199328_m1 | Guanosine monophosphate reductase 1 |
| 214431_at | GMPS | Hs00269500_m1 | Guanosine monophosphate synthetase |
| 200824_at | GSTP1 | Hs02512067_s1 | Glutathione S-transferase pi 1 |
| 202854_at | HPRT1 | Hs99999909_m1 | Hypoxanthine-guanine phosphoribosyltransferase |
| 204169_at | IMPDH1 | Hs01597683_g1 | Inosine-5'-monophosphate dehydrogenase 1 |
| 201892_s_at | IMPDH2 | Hs01021353-m1 | Inosine-5'-monophosphate dehydrogenase 2 |
| 209171_at | ITPA | Hs00738803_m1 | Inosine triphosphatase |
| 204168_at | MGST2 | Hs00182064_m1 | Microsomal glutathione S-transferase 2 |
| 201268_at | NME1-NME2 | Hs00897135_g1 | Nucleoside diphosphate kinase A and B |
| 205851_at | NME6 | Hs00195083_m1 | Nucleoside diphosphate kinase 6 |
| 243100_at | NT5C1B | Hs00403674_m1 | 5'-nucleotidase, cytosolic IB |
| 236703_at | NT5C2 | Hs01056741_m1 | 5'-nucleotidase, cytosolic II |
| 203939_at | NT5E | Hs01573922_m1 | 5'-nucleotidase, ecto (CD73) |
| 219708_at | NT5M | Hs00220234_m1 | 5',3'-nucleotidase, mitochondrial |
| 225367_at | PGM2 | Hs00217619_m1 | Phosphoglucomutase 2 |
| 201695_s_at | PNP | Hs01002926_m1 | Purine nucleoside phosphorylase |
| 209433_s_at | PPAT | Hs00601264_m1 | Phosphoribosyl pyrophosphate amidotransferase |
| 208640_at | RAC1 | Hs01902432_s1 | Rho family, small GTP binding protein Rac1 |
| 213603_s_at | RAC2 | Hs01036635_s1 | Rho family, small GTP binding protein Rac2 |
| 201802_at | SLC29A1 (ENT1) | Hs01085706_m1 | Solute carrier family 29 (nucleoside transporters), member 1, equilibrative nucleoside transporter 1 |
| 1560149_at | SLC29A2 (ENT2) | Hs00155426_m1 | Solute carrier family 29 (nucleoside transporters), member 2, equilibrative nucleoside transporter 2 |
| 203671_at | TPMT | Hs00909011_m1 | Thiopurine S-methyltransferase |
| 210301_at | XDH | Hs00166010_m1 | Xanthine dehydrogenase/oxidase |
