# Supplementary material for: Gene Expression and Thiopurine Metabolite Profiling in Inflammatory Bowel Disease – Novel Clues to Drug Targets and Disease Mechanisms?
Source: PLoS One. 2013 Feb 21;8(2):e56989. doi: 10.1371/journal.pone.0056989 (PMC3578787; doi:10.1371/journal.pone.0056989)
Supplement: Table S4 — Spearman rank correlations (microarray screening).a (DOC) [file pone.0056989.s005.doc]

**Table S4. Spearman rank correlations (microarray screening).^a^**

| **Selection basis: 6-TGN** | | | **meTIMP** | | **meTIMP/6-TGN** | |
| --- | --- | --- | --- | --- | --- | --- |
| **Gene** | **R_s_** | ***P*** | **R_s_** | ***P*** | **R_s_** | ***P*** |
| *HVCN1* | 0.75 | 0.0001 | -0.58 | 0.006 | -0.67 | 0.0008 |
| *TOX4* | 0.72 | 0.0002 | -0.35 | 0.12 | -0.53 | 0.01 |
| *SMAP2* | 0.70 | 0.0004 | -0.29 | 0.20 | -0.44 | 0.04 |
| *DEF8* | 0.69 | 0.0006 | -0.23 | 0.32 | -0.37 | 0.09 |
| *PLCB2* | 0.68 | 0.0007 | -0.56 | 0.009 | -0.69 | 0.0005 |
| **Selection basis: meTIMP** | | | **6-TGN** | | **meTIMP/6-TGN** | |
| **Gene** | **R_s_** | ***P*** | **R_s_** | ***P*** | **R_s_** | ***P*** |
| *UBE2A* | -0.74 | 0.0001 | 0.30 | 0.19 | -0.72 | 0.0002 |
| *FAM156A* | -0.74 | 0.0001 | 0.29 | 0.19 | -0.70 | 0.0004 |
| *CD1D* | -0.74 | 0.0001 | 0.42 | 0.06 | -0.74 | 0.0001 |
| *TUSC2* | -0.73 | 0.0002 | 0.33 | 0.14 | -0.67 | 0.0009 |
| *GNB4* | -0.72 | 0.0002 | 0.49 | 0.02 | -0.76 | 0.0001 |
| *MAP3K1^b^* | 0.72 | 0.0002 | -0.28 | 0.22 | 0.70 | 0.0004 |
| **Selection basis: meTIMP/6-TGN** | | | **6-TGN** | | **meTIMP** | |
| **Gene** | **R_s_** | ***P*** | **R_s_** | ***P*** | **R_s_** | ***P*** |
| *GNB4* | -0.76 | 0.0001 | 0.49 | 0.02 | -0.72 | 0.0002 |
| *FAR1* | -0.74 | 0.0001 | 0.64 | 0.002 | -0.62 | 0.003 |
| *LAP3* | -0.74 | 0.0001 | 0.47 | 0.03 | -0.65 | 0.001 |
| *CD1D* | -0.74 | 0.0001 | 0.42 | 0.06 | -0.74 | 0.0001 |
| *CTSS* | -0.74 | 0.0001 | 0.57 | 0.007 | -0.66 | 0.0012 |

^a^ The top five to six genes which showed the most significant Spearman rank correlations with the concentration of 6-TGN, meTIMP or the meTIMP/6-TGN concentration ratio, identified by microarray screening (n = 7325 probe sets).

^b^ The chromosomal alignment area of the probe set.
