# Supplementary material for: Gene Expression and Thiopurine Metabolite Profiling in Inflammatory Bowel Disease – Novel Clues to Drug Targets and Disease Mechanisms?
Source: PLoS One. 2013 Feb 21;8(2):e56989. doi: 10.1371/journal.pone.0056989 (PMC3578787; doi:10.1371/journal.pone.0056989)
Supplement: Table S5 — Spearman rank correlations (RT qPCR data vs. metabolite data)a of genes identified by microarray screening. (DOC) [file pone.0056989.s006.doc]

**Table S5. Spearman rank correlations (RT qPCR data *vs.* metabolite data)^a^ of genes identified by microarray screening.**

|  | **6-TGN** | | **meTIMP** | | **Ratio meTIMP/6-TGN** | |
| --- | --- | --- | --- | --- | --- | --- |
| **Gene** | **R_s_** | ***P*-value** | **R_s_** | ***P*-value** | **R_s_** | ***P*-value** |
| *CD1D* | 0.27 | **0.05** | -0.26 | 0.06 | -0.33 | **0.02** |
| *CTSS* | 0.16 | 0.26 | -0.39 | **0.003** | -0.38 | **0.004** |
| *DEF8* | 0.32 | **0.02** | -0.16 | 0.24 | -0.27 | **0.05** |
| *FAM156A* | 0.18 | 0.18 | -0.33 | **0.01** | -0.34 | **0.01** |
| *FAM46A* | 0.07 | 0.61 | -0.25 | 0.07 | -0.22 | 0.10 |
| *FAR1* | -0.04 | 0.75 | -0.15 | 0.28 | -0.13 | 0.33 |
| *GNB4* | 0.17 | 0.22 | -0.33 | **0.01** | -0.37 | **0.006** |
| *HVCN1* | 0.32 | **0.02** | -0.09 | 0.51 | -0.20 | 0.16 |
| *LAP3* | 0.24 | 0.08 | -0.22 | 0.10 | -0.28 | **0.04** |
| *MAP3K1_custom^b^* | 0.20 | 0.14 | -0.07 | 0.63 | -0.15 | 0.29 |
| *PLCB2* | 0.19 | 0.17 | -0.37 | **0.006** | -0.37 | **0.006** |
| *SLX1A* | 0.03 | 0.84 | -0.17 | 0.22 | -0.17 | 0.22 |
| *SMAP2* | 0.15 | 0.29 | -0.07 | 0.61 | -0.16 | 0.26 |
| *TGOLN2* | -0.10 | 0.46 | -0.17 | 0.23 | -0.11 | 0.43 |
| *TOX4* | 0.21 | 0.14 | -0.15 | 0.29 | -0.22 | 0.11 |
| *TUSC2* | 0.41 | **0.002** | -0.16 | 0.24 | -0.31 | **0.02** |
| *UBE2A* | 0.12 | 0.38 | -0.22 | 0.11 | -0.26 | 0.05 |

^a^ Spearman rank correlations between relative gene expression levels and the concentration of 6-TGN, meTIMP or the meTIMP/6-TGN concentration ratio in the expanded patient cohort (n = 54).

^b^ Assay designed based on the chromosomal alignment area of the probe set.
