# Supplementary material for: Gene Expression and Thiopurine Metabolite Profiling in Inflammatory Bowel Disease – Novel Clues to Drug Targets and Disease Mechanisms?
Source: PLoS One. 2013 Feb 21;8(2):e56989. doi: 10.1371/journal.pone.0056989 (PMC3578787; doi:10.1371/journal.pone.0056989)
Supplement: Table S6 — Spearman rank correlations (RT qPCR data vs. metabolite data)a of genes with a proven or potential relation to purines/thiopurines. (DOC) [file pone.0056989.s007.doc]

**Table S6. Spearman rank correlations (RT qPCR data *vs.* metabolite data)^a^ of genes with a proven or potential relation to purines/thiopurines.**

|  | **6-TGN** | | **meTIMP** | | **Ratio meTIMP/6-TGN** | |
| --- | --- | --- | --- | --- | --- | --- |
| **Gene** | **R_s_** | ***P*-value** | **R_s_** | ***P*-value** | **R_s_** | ***P*-value** |
| *ABCC4* | -0.25 | 0.07 | 0.07 | 0.62 | 0.15 | 0.27 |
| *ABCC5* | 0.12 | 0.41 | -0.12 | 0.39 | -0.15 | 0.27 |
| *ALDH1A2* | -0.14 | 0.32 | 0.11 | 0.43 | 0.11 | 0.44 |
| *CANT1* | 0.19 | 0.16 | -0.02 | 0.91 | -0.10 | 0.48 |
| *DPP4* | 0.08 | 0.54 | 0.20 | 0.15 | 0.14 | 0.33 |
| *ENTPD1* | -0.10 | 0.49 | -0.27 | **0.05** | -0.21 | 0.12 |
| *ENTPD5* | -0.07 | 0.61 | 0.03 | 0.84 | 0.06 | 0.66 |
| *GMPR1* | -0.06 | 0.65 | -0.01 | 0.93 | 0.03 | 0.84 |
| *GMPS* | -0.02 | 0.91 | 0.12 | 0.39 | 0.13 | 0.37 |
| *GSTP1* | 0.18 | 0.20 | -0.01 | 0.97 | -0.09 | 0.52 |
| *HPRT1* | -0.28 | **0.04** | 0.06 | 0.68 | 0.13 | 0.35 |
| *IMPDH1* | -0.02 | 0.88 | -0.16 | 0.26 | -0.14 | 0.32 |
| *IMPDH2* | -0.11 | 0.45 | 0.29 | **0.03** | 0.30 | **0.03** |
| *ITPA* | 0.11 | 0.44 | -0.05 | 0.71 | -0.08 | 0.57 |
| *MGST2* | 0.20 | 0.15 | -0.41 | **0.002** | -0.43 | **0.001** |
| *NME1-NME2* | 0.001 | 0.99 | -0.16 | 0.24 | -0.12 | 0.38 |
| *NME6* | 0.24 | 0.08 | -0.27 | **0.05** | -0.35 | **0.01** |
| *NT5C2* | -0.05 | 0.69 | -0.24 | 0.08 | -0.19 | 0.16 |
| *NT5E* | -0.09 | 0.51 | 0.42 | **0.002** | 0.42 | **0.002** |
| *NT5M* | -0.03 | 0.82 | 0.09 | 0.50 | 0.09 | 0.53 |
| *PGM2* | 0.09 | 0.50 | -0.20 | 0.15 | -0.21 | 0.12 |
| *PNP* | -0.09 | 0.50 | 0.10 | 0.49 | 0.14 | 0.32 |
| *PPAT* | 0.02 | 0.90 | 0.21 | 0.13 | 0.18 | 0.19 |
| *RAC1* | 0.27 | **0.04** | -0.24 | 0.08 | -0.37 | **0.006** |
| *RAC2* | 0.23 | 0.09 | -0.01 | 0.95 | -0.11 | 0.41 |
| *SLC29A1* | 0.23 | 0.09 | -0.12 | 0.37 | -0.20 | 0.15 |
| *SLC29A2* | 0.02 | 0.89 | 0.33 | **0.01** | 0.29 | **0.03** |
| *TPMT* | -0.12 | 0.38 | -0.45 | **<0.001** | -0.34 | **0.01** |

^a^ Spearman rank correlations between relative gene expression levels and the concentration of 6-TGN, meTIMP or the meTIMP/6-TGN concentration ratio in the expanded patient cohort (n = 54).
